# Clinician Experiences at the Frontier of Pharmacogenomics and Future Directions

**DOI:** 10.3390/jpm15070294

**Published:** 2025-07-07

**Authors:** Stefan Thottunkal, Claire Spahn, Benjamin Wang, Nidhi Rohatgi, Jison Hong, Abha Khandelwal, Latha Palaniappan

**Affiliations:** 1 Department of Medicine, Stanford University School of Medicine, Palo Alto, CA 94305, USA; stefan01@stanford.edu (S.T.);; 2 Department of Pharmacy, Stanford Heath Care, Palo Alto, CA 94305, USA; cspahn@stanfordhealthcare.org (C.S.);; 3 Division of Hospital Medicine, Department of Medicine, Stanford University School of Medicine, Palo Alto, CA 94305, USA; 4 Division of Immunology & Rheumatology, Department of Medicine, Stanford University School of Medicine, Palo Alto, CA 94305, USA; jison@stanford.edu

**Keywords:** pharmacogenomics, implementation, pharmacology, genetics, adverse drug reaction

## Abstract

Pharmacogenomics (PGx) has emerged as a powerful tool to personalize drug selection and dosing based on a patient’s genetic profile. However, there are a range of challenges that impede uptake in current clinical practice. For example, clinicians often express frustration with commercially available PGx panel tests, which fail to consistently include all key actionable PGx genes (according to the Clinical Pharmacogenetics Implementation Consortium (CPIC), Food and Drug Administration (FDA) PGx guidelines, or The Dutch Pharmacogenetics Working Group (DPWG) guidelines) and instead are too long with clinically unimportant information (unvalidated genotypes). Additionally, the lack of EMR integration, clinician education and awareness of the benefits of PGx impedes uptake. This paper examines key challenges identified in clinical practice and proposes future directions, focusing on limiting PGx reports to essential data, providing point-of-prescription alerts, and establishing reimbursement pathways that encourage adoption. Future directions include leveraging large language models, integrating point-of-prescription alerts and phenoconversion calculators into the electronic medical record, increasing the genomic diversity of PGx study populations, and streamlining coverage by payers.

## 1. Introduction

Pharmacogenomics (PGx) integrates concepts from genomics and pharmacology to address individual variations in drug response. This is particularly relevant in the context of polypharmacy, where PGx can help tailor treatments and reduce adverse drug reactions, especially in vulnerable populations with chronic disease. These patients typically require lifelong treatment with prescription medications which contributes significantly to high health care costs [1,2,3,4]. Optimization of therapy for high-risk patients—particularly in transplant medicine, cardiology, neurology, oncology, geriatrics, and other high-acuity settings—can greatly benefit from PGx insights. Implementation in general practice settings is underutilized but has been researched with relatively more depth, demonstrating strong value potential in optimizing common drugs such as statins, anti-coagulants, beta blockers, and opioids [5,6,7,8,9]. These clinicians also manage many elderly patients with polypharmacy who can especially benefit from PGx. While pharmacogenomic-guided therapy is cutting-edge, the practical path to implementation requires addressing age-old challenges that are common when incorporating new clinical evidence and therapies into real-world clinical practice.

The prevalence of patients carrying at least one actionable pharmacogenetic variant is estimated to be greater than 95% [10,11]. Current prescribing practices do not incorporate PGx and instead opt for a trial-and-error approach (i.e., patients are prescribed medications based on population-based studies and then monitored for adverse drug events (ADR) and efficacy. Change in medication for an individual patient is considered in the event an ADR or other adverse outcome occurs). This leads to increased morbidity and poor patient experiences and presents an opportunity to optimize prescribing. A recent study identified that 31% of patients within the hospital-based sample were prescribed at least one drug for which they had a high-risk variant necessitating a drug dosage change or alternative prescribing according to international guidelines [10]. These findings mirror that of several studies across ambulatory care and hospital settings, which find that the rate of patients experiencing suboptimal prescribing (where alternate medications are recommended due to PGx interactions) is 31–40% [5,12,13].

Despite the growing evidence supporting the need to implement PGx-based prescribing, clinicians face barriers implementing the recommendations put forward by PGx reports due to a lack of intuitive design, complicated report structure, irrelevant genes, and lack of integration with electronic medical records (EMRs). As a team we have reflected on our experiences and seek to share anecdotes and illustrative examples. In this article we share our experiences implementing PGx into medical consultations provided in ambulatory care and hospital settings and demonstrate the mismatch between the promise and real-world clinical implementation of PGx. We hope to highlight the opportunities to refine PGx tools to make them more actionable at the point of care.

## 2. Current Implementation Challenges with PGx

**Figure 1:**
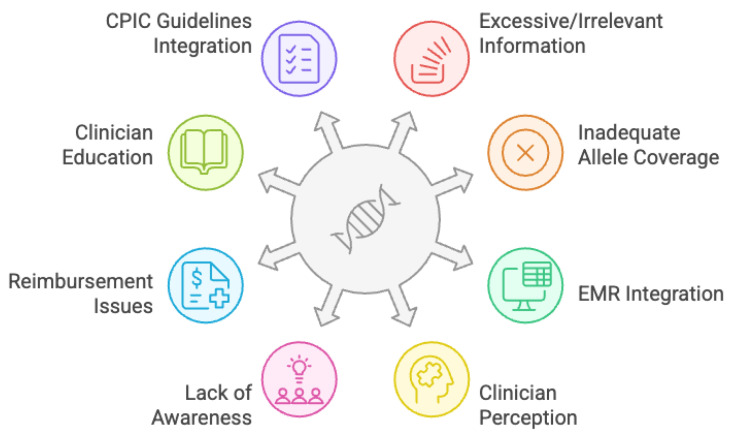
Current implementation challenges with pharmacogenomics.

We have identified eight key challenges facing real-world clinical implementation of PGx based on our experiences of implementing PGx in ambulatory care and hospital settings at Stanford (using an external lab testing provider, see Figure 1). These reflections are informed by our involvement in implementation activities and observation of barriers encountered during clinical workflow integration.

We acknowledge the limitations in generalizing these barriers to other clinical ecosystems and specialties such as general practice. However, we believe many of these issues such as poor clinician awareness and education, and reimbursement, reflect systemic gaps in the US context:

1. Integration of Clinical Pharmacogenetics Implementation Consortium (CPIC) guidelines;
2. Inadequacies in allele coverage and consequent metabolizer categorization;
3. Excessive and irrelevant information and clinician palatability;
4. Integration within EMR systems;
5. Perception of benefits of PGx by clinicians;
6. Lack of awareness of positive patient experiences from engaging in PGx;
7. Reimbursement.
8. Clinician education

### 2.1. Integration of CPIC Guidelines

The Clinical Pharmacogenetics Implementation Consortium (CPIC) publishes guidelines that map genotype to phenotype with recommendations for drug selection and dosing. This is true for other guidelines produced by key consortiums and bodies such as the Dutch Pharmacogenetics Working Group, U.S. Food and Drug Administration, and Association for Molecular Pathology. For simplicity, we focus our article on omission of crucial drug–gene pairs reported in CPIC guidelines in commercially available PGx panels [14,15]. One clinician reported that the PGx vendor they were using had failed to test for RYR1/CACNA1S genes, variants of which may be associated with a rare but life-threatening condition called malignant hyperthermia and would have been relevant for surgical patients needing general anesthesia. On the other hand, there are pharmacogenes with evidence of association but lack of inclusion within guidelines due to the time it takes to compile evidence. For example, N-acetyltransferase 2 (NAT2) and its interaction with amifampridine is a pharmacogene with significant evidence for clinical utility, and FDA warnings, yet is not featured on CPIC guidelines [16,17]. Incomplete coverage imposes a significant barrier to clinicians who rely on these guidelines for actionable insights.

Comparing a single commercial pharmacogenetic testing report and recommendations for antidepressants within CPIC guidelines, one study identified 12.8% genotype-to-phenotype translation discrepancies [18]. Of the 266 evaluated medication recommendations, there were 114 (42.9%) discrepancies across the two pharmacogenetic testing companies tested [18]. Similarly, a retrospective chart review demonstrated that the prevalence of conflicting phenotype translation was 28.8% for *CYP2D6* and 32.2% for *CYP2C19* genotypes when CPIC guidelines were compared against the commercial lab outputs [19].

These discrepancies undermine provider trust in commercial PGx vendor reports and add the need for an additional provider to ensure recommendations are aligned with CPIC or another preferred guideline. This results in confusion for the patients who may receive PGx reports directly from the commercial vendor or lead to added personnel costs and delay in optimizing medications for the patients based on PGx results. Omission of reporting crucial drug–gene pairs that are clinically important for the patient deprives them from potential benefit.

### 2.2. Inadequacies in Allele Coverage and Consequent Metabolizer Categorization

Another separate issue is breadth of allele coverage, as explained by Nicholson et al. [20]. A PGx test is only as good as the design and variants included. Currently, the testing of variants by commercial vendors can range from targeted genotyping to full-gene sequencing. There is lack of standardization for some alleles and poor lab provider adherence to some of these standards regarding the minimal set of alleles that should be tested. Our provider fortunately has included all recommended minimum alleles following our vetting process. However, for other providers, this is a critical gap considering the differences in the frequency of these alleles across different populations and ethnicities [21]. When certain alleles are not tested, PGx results may misclassify patient’s metabolizer status and misguide clinical decisions. For example, CYP2D6 *4 allele is most commonly attributed with poor metabolizer status and should be included in the PGx assay [22]. This allele is found in 18.5% of people of European ancestry and in 3–5% of those of African ancestry [22].

Another example would be lack of detection of CYP2C9 *8 allele in a patient of African ancestry who is being prescribed warfarin. This allele can result in reduced function [20]. If this allele is included in the PGx panel, a patient with CYP2C9 *1/*8 genotype would be categorized as an intermediate metabolizer but if this allele is not included and tested in the PGx panel, this patient would be reported as CYP2C9 *1/*1 genotype and be erroneously categorized as a normal metabolizer [20].

Therapeutic decision making based on these results can have significant clinical consequences, for example, providing warfarin at standard doses to a patient when the true genotype factoring the *8 allele would categorize this patient as a poor metabolizer [20]. It also undermines the confidence in the decision making process for clinicians and highlights the risks of PGx tailored care which may use data with limited representation of rarer alleles present in ethnically diverse populations [23,24].

Currently some vendors report PGx medication interactions with the relevant genotype and specific metabolizer phenotype as justification. For example, a summary at the top of the report saying major gene drug interaction for atorvastatin (SLCOB1 PM) Where the medicolegal risk falls if clinicians adopt the recommended prescribing action (without awareness of allele gaps), resulting in adverse events, is complex and unclear to us. The consequences of a case like this in the future, could be reduced provision of medication specific recommendations in vendor provided reports, resulting in more test interpretation burden for clinicians. However, while multiple clinicians may prescribe medications to patients, only a few clinicians may have the required knowledge to accurately interpret genotypes or identify the gaps in testing. Access to PGx-trained pharmacists may be even more limited. Without having medication specific recommendations integrated into PGx reports, the potential benefits to patients become far more limited and increases the cognitive burden and potential medicolegal risk for the prescribing clinicians.

This concept of listing medication specific interactions in gene reports faced challenges previously when they were patient facing. The FDA has enforced regulations surrounding marketing to prevent patients tailoring medications themselves based on panel results-risking them stopping medical treatment or inappropriately selecting alternate drugs [20,25]. In fact, the FDA cleared 23andMe to perform PGx tests and report genotypes and phenotypes, but not medication recommendations, directly to consumers in 2018 [20]. Although clinicians have more medical literacy and capacity to understand the nuanced pros and cons of making medication changes, they may lack the knowledge to engage with more complex PGx concepts. These may include phenoconversion and allele gaps, which necessitates deeper understanding than medication binning (severe/moderate/no interaction). A key question remains: Should clinicians be responsible for recognizing allele gaps, or should testing companies be required to expand allele coverage, even if it increases costs?

To address allele gaps, the Association for Molecular Pathologists has a project underway to recommend a minimum set of alleles that laboratories should test for, with current recommendations including CYP3A4, CYP3A5, TPMT and NUDT15, CYP2C19, CYP2C9, CYP2D6, and genes important for warfarin testing [20]. This initiative aims to standardize testing and reduce variability in PGx panel quality, which is critical for clinical trust and adoption. However, in future, the field would benefit from FDA mandates or oversight over labs to ensure key alleles are tested. Although recent court rulings suggest that this is not within the FDA’s purview to regulate, thus the relevant regulatory authority must be identified to take lead [26,27].

For the current state of practice, we believe to overcome these risks, PGx reports need to flag potential gaps in alleles included in panel tests and raise awareness of these gaps for clinicians when genotype/phenotype discordant results occur. Given the time constrains clinicians operate within, it is unreasonable to expect clinicians to be aware of all key alleles per cytochrome. Although simplified reports and clinical notes formatted as metabolizer type/drug recommendations are crucial for streamlined decision making, they cannot describe this nuance, thus health providers need to flag this limitation when providing advice. Alternatively, institutions or PGx clinicians could review lab test’s coverage to ensure that they are comfortable with the standard before adoption/basing recommendations on the test results [28]. They can then choose to utilize only test vendors who they have vetted [28].

### 2.3. Excessive and Irrelevant Information and Clinician Palatability

Another recurring challenge we have identified at Stanford is that many PGx reports include details that clinicians neither need, nor can readily apply. For example, listing pharmacogenes for drugs that are very rarely prescribed or filled with genotypes that are in development or not yet validated, or are not supported by guidelines. The extraneous information can distract from core therapeutic decision points, leading to confusion and reluctance to utilize PGx results. While in-house tests can be customized to test the most relevant genes or produce more focused reports, the majority of health care providers or institutions lack the capacity and resources to develop in house testing.

*“MTHFR is a gene with conflicting evidence, and yet the test provider that we use reports on it. This adds to the bulk of PGx reports and makes it harder to extract the most crucial information for patients”*

This frustration calls for tighter curation of PGx reports, ensuring tighter regulation of reports to include only PGx evidence-supported drugs and genes, using relevant guidelines published by established organizations/consortiums.

A study investigating pharmacist’s vs. general practitioner’s preferences regarding PGx service design found that among both groups, respondents preferred to receive pharmacogenetic results via a “narrow” gene panel, rather than a “focused” single result or a “broad” panel [29]. Although broad panels are more cost effective, a challenge that may arise is when a specialist orders PGx testing and it discovers actionable modifications for medications prescribed by another specialist. Communicating these findings to the other specialist requires a. explaining the significance of PGx to a clinician who may not be as invested in applying learnings, and b. awareness of whether clinically suitable alternatives exist, and if communicating those findings to them is beneficial/would result in a change to their prescription.

Clinician Palatability: The Lack of conciseness in PGx reports

Clinicians operate in high-pressure environments, managing a high volume of patients and need clear, relevant, and concise information on demand. PGx reports that have been “trimmed down” to the essential gene-drug pairs provide a quick, chart-ready review of genomic information, and this strategy has been adopted at Stanford. Within the literature, clinicians have reported that PGx reports come back in an unreasonable “35-pages long” report, which is highly time consuming and unintuitive to read through. “There is a big need for a concise report, with clear directions or actions” [7].

The core concept is to offer a single-page summary highlighting the genotype of known clinical impact and their recommended interventions, particularly for high-risk or commonly prescribed medications (e.g., warfarin, clopidogrel, certain opioids). This prevents the clinician from needing to trawl through an extensive genotype/phenotype list, as currently provided by commercial entities, to conduct PGx informed prescribing [30]. This can be combined with EMR integrated alerts to provide flagging opportunities when initiating prescriptions, and a report to fall back on for further clarity and context. Adopting conventions from echocardiogram reports in cardiology, PGx guidance can be formatted in terms of didactive or directive statements in such reports.

**Directive statements** focus on providing specific recommendations and actionable insights for clinical management based on findings.
**Findings:** ▪“The patient is identified as a poor metabolizer of CYP2D6 based on genetic testing.” **Recommendations:** ▪“Avoid prescribing codeine and tramadol due to the risk of inadequate analgesia and potential adverse effects.”▪“Consider alternative pain management strategies, such as using non-opioid analgesia or non-CYP2D6 dependent opioids (e.g., hydromorphone, fentanyl).”▪“Monitor the patient closely for pain control and side effects and adjust the treatment plan as necessary.” 

**Didactic statements** aim to educate clinicians about specific explanations and context regarding PGx findings, providing general guidance.
**Findings:** ▪“Genetic testing has revealed that the patient is a poor metabolizer of CYP2D6”. **Explanation:** ▪“CYP2D6 is part of the cytochrome P450 family of enzymes, which play a critical role in drug metabolism. It is responsible for converting certain opioids, such as codeine and tramadol, into their active forms, and variations in its gene can significantly affect how individuals respond to medications, including opioids.”▪“As a poor metabolizer, the patient may experience reduced efficacy of these medications, leading to inadequate pain relief. Additionally, there is a risk of accumulation of the parent drug, which can increase the likelihood of side effects.” **Recommendations:** ▪“Given the patient’s genotype, it is advisable to avoid codeine and tramadol. Analgesics that do not require CYP2D6 for activation should be considered.” (provide alternatives

### 2.4. Integration Within EMR Systems

Many clinicians report challenges with adding PGx panel results to the EMR [7]. Some clinicians opt to write their medication-specific recommendations based on patient’s PGx panel as a patient note in the EMR or add their recommendations under “allergies” or the “problem list”. Adding the information under allergies serves as a definite reminder or alert when a relevant medication is being prescribed by anyone across the institution although some of these alerts may not always require a change in the management plan. Integration of alerts within the electronic medication administration record has been explored within the literature and will be analyzed in further detail in Section 3.2.

In Epic EMR, the tab on genomics provides a defined location for this data to be saved. However, many clinicians may not be aware of this section, or routinely access this tab in their workflow, or not have access to this section of Epic across the ambulatory, hospital, or emergency department settings where medications may be prescribed. This tab may or may not be readily available to clinicians and pharmacists outside a certain institution. The integration of PGx results in the EMR may or may not strike the optimal balance of simplicity vs. relevant clinical actionability and ensure automatic transfer of results from PGx reports provided by commercial vendors.

A key concern raised in a Mayo Clinic clinician focus group, is what would happen to test results when patients are cared for by external providers? Systems that do not have access to the same EMR system or e-consult services. Some patients may have traveled for care and be going back to their local primary providers, clinicians who may be in rural areas with resource limitations. Thus, focusing exclusively on EMR integration alone would be insufficient in addressing this barrier.

### 2.5. Awareness and Characterization of Benefits of PGx Among Clinicians

We have identified that to some clinicians, PGx testing is perceived negatively often due to inconsistent gene inclusion, lack of perceived patient benefits or inclusion in clinical practice guidelines. Such perceptions may limit the broader community’s willingness to adopt PGx based interventions, especially if they correlate with poor reimbursement, complicated implementation pathways, or questionable return on investment.

Often the value of PGx integration is conceptualized in terms of screening rare adverse effects. To prevent severe reactions such as Steven Johnsons Syndrome or Toxic Epidermal Necrolysis following Allopurinol (HLA–B*58:01) or Carbamazepine (HLA-B*15:02) [31]. This is a reductionist view of PGx and deprives appreciation of the patient wellbeing and medication efficacy and side effect reduction benefits. Additionally, as a measure it can lead to underestimation of the benefits of PGx at a population health level. As the prevalence of some of these conditions are low, the utility of testing may be perceived to be zero among clinicians (due to their limited first-hand sample size) [32]. Out of 50 tests, none may benefit as the patient must have both the genotype (which could have an allele frequency of 5%) and also be prescribed a genotype disconcordant drug at the time [32].

Furthermore, a significant body of PGx research has focused on warfarin prescribing (VKORC1), which has a contested application in practice due to the complex interplay between non-genetic factors affecting warfarin dosing, urgent nature of many warfarin indications being incompatible with PGx result turnaround time, already tailored dosing regimen using INR, and now increasingly rare use since the introduction of direct oral anticoagulants [33,34].

Opioids are commonly prescribed after surgical procedures. For many patients, surgery is the first time they are prescribed opioids. Individualized prescription of opioids to surgical patients based on their personal opioid metabolism profile through PGx can help reduce ADRs, risk of opioid use disorder, and improve analgesia The CPIC guidelines provide clear recommendations for tramadol and codeine and CYP2D6 genotype [35]. However, codeine is rarely prescribed for management of postoperative pain and tramadol is sometimes prescribed. There are no PGx recommendations for the more commonly prescribed opioids for management of postoperative pain such as hydrocodone, oxycodone, hydromorphone, fentanyl, morphine, or methadone. The OPRM1 A118G polymorphism has been associated with variation in analgesic response to postoperative opioids, opioid induced respiratory depression, and opioid addiction. A meta-analysis including 4607 patients reported that carriers of G-allele did not experience adequate analgesia at standard doses of opioids and needed higher doses of opioids [36]. These effects were noted to be strongest in Asians (vs. Caucasians), with morphine use (vs. fentanyl), and those were visceral (vs. somatic) pain. The CPIC guidelines, however, report there is insufficient evidence to make any recommendations for OPRM1 variants and opioid prescription. Thus, US evidence on benefits of PGx guided opioid selection is limited as applying current CPIC guidelines results in limited actual prescribing changes in real world settings [37]. On the other hand for non-opioid medications for pain control, non-steroidal anti-inflammatory drugs are commonly prescribed and CPIC guidelines provide clear recommendations for CYP2C9 genotype [38].

The benefits of PGx panels extend beyond these few drugs, and the cost per genotype is decreased in larger panels, increasing utility and cost/benefit ratio [2]. Moreover, the genotypes uncovered by a PGx panel are valid for many years and the benefits compound as patients age and are prescribed more medications. Thus, clinicians must be aware that PGx testing is more of a long-term investment, regardless of whether they immediately see genotype disconcordant drugs or resultant prescription changes. This also highlights an important point that instead of checking for individual genotypes, which is current state for several conditions (HLAB5801 for allopurinol in patients with gout, TPMT for azathioprine in patients with vasculitis and lupus patients), we should adopt more extensive PGx panel testing at diagnosis of certain chronic conditions (gout, vasculitis, lupus), for which several medications are likely to be prescribed during the course of their disease including the need for individual genotypes above. In patients with normal metabolizer status for the relevant genotypes, having a PGx panel can provide reassurance to the patient, family, and the prescribing clinician that they have reviewed and incorporated this additional consideration in their clinical decision making process.

In a clinician’s account, PGx was reported to have “the potential to impact future care. PGx testing has identified genotype abnormalities affecting antiplatelet metabolism so it was listed as a drug allergy in case the patient develops a heart attack requiring interventional cardiology procedures in the future for which they would be prescribed this particular medication”

Regarding validity of results long term, although the patient’s genotypes do not change, the number of variants discovered and evidence supporting their recommendations increases over time. As of September 2019, CPIC had published 23 guidelines, covering 16 genes, 46 drugs, however currently in February 2025, CPIC has published 28 guidelines across 34 genes and 164 drugs [39,40].

#### Optimizing prescriptions to improve efficacy and minimize risk of ADRs

Reframing the value of PGx panel testing as a tool to optimize treatment efficacy and minimize ADRs is crucial. For instance, picking alternative cholesterol lowering medications instead of statins first line for patients with poor metabolizer status (SLCOB1) to reduce risk of statin induced myopathy [5]. A recent Lancet randomized control trial totaling approximately 7000 patients, identified significantly reduced ADRs in the intervention group: 21.0% vs. 27.7% in the control group for patients with actionable variants [41]. This study examined a wide range of specialties, drugs, and clinical environments and only examined 12 genes yet still produced a number needed to treat (NNT) value of approximately 15 [41].

For prescription of antidepressants, a recent meta-analysis identified those who received PGx-guided medications were 41% to 78% more likely to achieve remission (NNT = 24) and 20% to 49% more likely to respond to antidepressants than patients receiving treatment-as-usual (NNT = 21) [42].

### 2.6. Lack of Awareness of Positive Patient Experiences from Engaging in PGx

Our clinical experiences have identified positive patient perceptions towards PGx; however, we believe there is a lack of awareness of these benefits among the broader clinical community. Improved awareness of positive patient experiences could enhance clinician engagement and willingness to integrate PGx into practice. Anecdotally, patients feel a sense of trust after PGx testing, feeling that the doctor is providing personalized care and has carefully incorporated the patient’s perspectives and anxiety about potential adverse effects or lack of efficacy of the medication after a thorough review of not only the medication and the current evidence, but also the potential drug–drug, drug–gene, and drug–drugdrug–gene interactions. PGx sometimes helps provide a reason to the patient (and clinician) why a particular medication caused unexpected adverse effects (e.g., severe respiratory depression after a low dose of tramadol in CYP2D6 ultrarapid metabolizer) or was not as effective as expected for a certain patient (e.g., recurrent stroke or myocardial infarction despite taking clopidogrel in CYP2C19 poor metabolizers) although it worked well for many others and is recommended in clinical practice guidelines. Some patients get labeled as “drug seekers” if they request more than the standard doses of an opioid, or “lightweight” (i.e., being “over-sensitive” to even low doses of opioids), or “picky” if they report that only certain opioids work for them. PGx can help remove such cognitive biases and stigma and provide a scientific rationale for differences in effectiveness of pain or nausea medications for patients with certain ancestries.

A PGx intervention targeting medically underserved patients found that patient treatment satisfaction significantly increased over the 6 months after PGx testing (59.9 ± 16.9 vs. 64.4 ± 13.6, *p* = 0.001) [11]. According to another study, privacy, empathy, medical decision making, and a composite personalized care score, were found to have greatly increased post visit when physicians considered PGx test data. Crucially, personalized care scores were significantly greater after physicians used PGx results to make medication changes (4.0 vs. 3.0, *p* < 0.001) in comparison to prescribing visits without PGx guidance [43].

In clinical practice, providers have reported patients feeling that PGx guided treatment “helped validate their personal experiences with medications”, including “explaining their sensitivity to side effects from beta-blockers (CYP2D6 IM) or not achieving desired analgesic effects from opioid medications (CYP2D6 UM)”

#### Medication Adherence

Aligning with our experiences, a study found that among participants who reported imperfect drug adherence, most (91%) stated they would be more likely to use their medication as prescribed if PGx results guided drug or dose selection [44].

However, studies on drug adherence outcomes following PGx guided care is limited, and they often suffer from small sample sizes. Some papers have demonstrated increased adherence using PGx [8,45]. A study on antidepressants adherence for treatment of major depressive disorder found significantly increased rates of adherence which was sustained at 6 months (38.1% in the PGx tailored group vs. 24.8% in the treatment as usual group; *p* < 0.001) [46]. Another study focused on underserved communities found that medication adherence significantly increased over the study period [11]. A primary care study found that PGx testing increased 6-month statin adherence by 18.4% [45]. Another study focused on statins found over double the prescription filling rate post PGx guided care (55% versus control at 20%, *p* < 0.001), and also identified that the act of genetic testing itself was a driver, as even non-carriers of risk alleles showed improved adherence [8]. The authors hypothesized this was due to reassurance about lower ADR risks [8].

On the other hand another study found no global difference in medication adherence following PGx guidance, although the study noted that of the patients who had been prescribed high-risk PGx medications, those with PGx-drug incompatibility were more than twice as likely to have low medication adherence, compared to those genetically tailored prescriptions (OR 2.4 (1.03–5.74), *p* < 0.05) [47]. This highlights that PGx optimization of medications can result in treatment regimens that some patients are more likely to adhere to. Whether the mechanisms for improved adherence are more psychological (perceived increased care quality and trust) or physiological (improved drug efficacy and tolerability due to gene concordant prescribing), remains an open question. Regardless, perhaps as public PGx awareness and the PGx literature grows, positive psychological attitudes toward personalized care could reinforce PGx’s role in improving medication adherence.

### 2.7. Reimbursement

Reaffirming our clinical experiences, several authors have cited cost as a major barrier to PGx implementation (costs of testing, interpretation, and follow-up care) [9,11,48,49]. Overall reimbursement rates for PGx tests range from 36% to 48% across payers according to a 2023 study [50]. PGx panels are reimbursed at a significantly higher rate (74%) compared to single-gene tests (43%) [50]. Medicare coverage for PGx testing has expanded since 2020, with most Medicare Administrative Contractors now approving comprehensive coverage. Despite these efforts, more work is needed to expand coverage.

The economic value of PGx panel testing is well justified, with a recent systematic review finding that 71% of studies evaluating the cost of PGx testing for drugs with CPIC guidelines concluding that testing was cost-effective or led to cost-savings [51]. Multigene testing strategies and preventative PGx testing instead of reactive, demonstrated greater cost effectiveness [51]. An economic modeling study focused on major depressive disorder found that based on ADR costs, hospitalization costs in the psychiatric clinic, cost of psychiatric medications, follow-up costs, while considering genetic testing cost and therapist sessions, a 48.5% treatment cost reduction was identified for participants in the PGx-guided group, with an additional slight improvement in the quality of life (0.935 versus 0.925 QALYs) [52].

Clinicians face key reimbursement barriers including inconsistent insurer coverage and limited CPT codes [53]. PGx testing coverage is not complete with most health insurers covering 10 or fewer drug–gene pairs according to a paper published in February 2025 [15]. Medicare reportedly featured the greatest number of single drug–gene pairs covered (65), followed by Avalon Healthcare Solutions (50), UnitedHealthcare (45), and Centene (33) [15]. Despite panel tests being cited as more cost-effective within the literature, insurers define eligibility based on unique criteria (for example, UnitedHealthcare reimburses panel testing only for those with a diagnosis of major depressive disorder or generalized anxiety disorder [15]. Drugs impacted by *HLA*-based hypersensitivity reactions and/or those that feature FDA boxed warnings (with the exception of codeine and tramadol) tend to have more coverage [15].

Clinicians have reported complexity in ordering PGx panels for patients. A general issue is knowing patient eligibility criteria for insurance coverage of PGx tests, such as failing prior treatment according to payor guidelines or taking high risk medications [7]. For panels specifically, we have noted that patients need to have applicable medications/indications for 2 different genes at a minimum. This requirement disincentivizes pre-emptive panel testing, which is a more cost-effective strategy, in favor of reactive testing [51].

For patients who have previously undergone any PGx testing, a key policy directive for payors is that reimbursement cannot be granted for a gene if it has already been genotyped. This presents confusion for panels, as although the clinical intention is to identify a broad spectrum of relevant genotypes, the incidental inclusion of the repeated gene may go against reimbursements directives.

In the future as reimbursement models evolve, guidelines themselves may require reassessment to focus on the most payoff-rich gene-drug pairs and to define the boundaries of routine screening. A 10-point checklist to systematically assess cost/benefit to evaluate implementation of specific PGx tests has been devised by Sanghvi et al. [54]. This checklist recognizes the importance of considering operational challenges, defining target cohorts, evidence of improvements in real world patient outcomes, and evaluation considerations.

Another key issue regarding reimbursement is insurance coverage for alterative drugs based on PGx profile. It was reported by one clinician that *“Insurance companies do not change coverage of medications based on PGx report findings. For example, a patient with positive HLAB5801 and tophaceous gout could not start febuxostat since that is not covered by insurance.”*

### 2.8. Clinician Education

Even with broad scale availability of panel testing, a key bottleneck is clinician education (e.g., physicians, advanced practice providers). Access to PGx-trained pharmacists may also be limited, especially at the time of prescription. This may be true in both community settings and academic medical centers. Even if testing is performed at one institution and medications prescribed in accordance, to have a meaningful impact for a particular patient, all the prescribing clinicians in every subsequent clinical setting need to appreciate why certain medication recommendations were made based on PGx during hand-offs and have an understanding of PGx and phenoconversion for application to future prescriptions or for dose titrations.

Physicians, pharmacists, and nurses report limited knowledge about PGx and its use in patient care [55]. In a survey of US pediatricians, only 7.2% were aware of CPIC pharmacogenetic practice guidelines and only 38.5% were aware that some drugs had FDA drug labels with PGx information to guide treatment [56].

The literature reports a wide range of causes for this lack of awareness, including lack of inclusion of PGx in curriculums, low availability of continuing education modules, competing physician priorities leading to limited time for education, and concerns surrounding evidence of clinical utility [55,57,58]. A study of medical and pharmacy students in the United Arab Emirates found that over 59.7% felt they did not receive enough knowledge or sufficient training and 58.7% agreed that currently there was poor guidance in how to apply PGx in clinical practice [58].

A study conducted in the United States reported that physicians with five or less years of practice experience favored genetic testing and were more prepared and confident using genetics in the clinical decision making process than physicians with more than five years of practice experience [59]. Clinician awareness of resources was also identified as a key barrier. Only 23% of clinicians in the study felt they could find and use trusted sources of information to comprehend and communicate genetic based risk to patients [59].

Given this gap in reliable resources, consulting resident pharmacists in-house can be an invaluable resource to clinicians to help clarify reports and answer any questions. This is especially beneficial for sole practitioners who might not have access to PGx trained professionals or other resources. However, it must be noted that the responsibility of PGx knowledge cannot fall on pharmacists completely. A view which emerged from a clinician focus group study from the Mayo Clinic was, “Why should we learn about PGx if we could just refer them to the pharmacist anyway?” [7]. Not only does this view fail to appreciate the low uptake of PGx in Pharmacy school curriculums, but also the fact that clinicians act as the gatekeepers for PGx—if they do not recognize when it is needed and refer patients, no one else will. Clinicians need to have at least a certain baseline level of PGx knowledge regarding key PGx drugs, identifying PGx discordant symptoms, and test ordering. Furthermore, even if you obtain input from pharmacists, the clinician ultimately bears the responsibility to make the clinical decision and therefore also needs to have a foundational understanding of how to interpret PGx.

## 3. Future Directions

In this section, we highlight 5 key opportunities to refine PGx tools to make them more actionable at the point of care.

1. Leveraging Large Language Models (LLMs)
2. Clinical Decision Support (CDS)
3. Phenoconversion calculators and combined genotype prediction models
4. Increased genomic diversity and PGx integration into drug development pipelines
5. Prioritization of High Yield Fields and Groups

### 3.1. Leveraging Large Language Models (LLMs)

Recent advances in artificial intelligence, particularly large language models (LLMs), offer an opportunity to automate the interpretation of complex genomic data. LLMs can serve as a backbone for real-time analytics, rapidly translating genotype-phenotype correlations into succinct clinical guidance. Where guidelines or evidence are limited, LLMs can highlight uncertainties and direct clinicians to the most current references, including evidence-based guidance from entities like CPIC.

A key theme identified in a focus group of clinicians was a desire for AI risk prediction tools that could automatically find patients who could benefit from a PGx test. In addition to identifying patients, they also wanted tailored alerts to the specific patient to increase the usefulness of alerts and reduce alert fatigue [7].

Additionally, LLMs can provide robust ability to PGx reports to relevant patient medical history (providing oncology focused list for patients with lung cancer). Its ability to expand results and address queries opens opportunities for clinicians with limited knowledge of PGx to rapidly cross check their prescribing decisions. Furthermore, they can serve as valuable patient education tools to explain results and facilitate shared decision making.

These tools could also address allele gaps in PGx panels through leveraging datasets and crosschecking to identify potentially missed alleles. Such a tool could be characterized as a genotype disconcordant genotype/phenotype response investigator and hypothesize potential missed alleles responsible through leveraging patient demographics. In addition, utilize medical history and medication list to theorize phenoconversion mechanisms/contributors. In future, LLMs could be used to conduct literature reviews to provide patient tailored risk stratification for individuals with particular phenotypes and comorbidities.

At this time, an LLM pharmacogenomics interpretation tool we have developed has demonstrated promise in translating genotypes into phenotypes according to CPIC guidelines and providing patient tailored PGx treatment recommendations. This has huge implications in addressing the lack of concordance with commercial genotype to phenotype translations + medical recommendations vs. CPIC guidelines, and easing burdens in manual report interpretation.

### 3.2. Clinical Decision Support (CDS)

A more targeted approach is needed when implementing PGx into the prescription workflow. Providers stated that while results were, in general, easy to access and understand, the biggest challenge was that remembering to access PGx results was not yet a part of their normal clinical workflow [60]. In one study, it was reported that a patient had been prescribed duloxetine despite the fact that it was noted as having a severe gene–drug interaction according to PGx results [61]. Critically, the provider did not document a reference to the previously completed PGx test results when making the decision to prescribe the drug. This finding raises the issue that although PGx results may be present in patient files, providers may not remember to check the results in future prescribing decisions [61].

Instead of requiring clinicians to comb through lengthy PGx reports, point-of-prescription alerts can inform them of potential genotype-driven contraindications or dose modifications [62,63]. For instance:

• Prescribing clopidogrel: The system might trigger a real-time notification recommending an alternative such as ticagrelor if the patient carries a CYP2C19 loss-of-function allele.
• Prescribing a tricyclic antidepressant: The system could suggest dose adjustments or an alternative drug if a CYP2D6 variant is present.

Such targeted, automated alerts reduce cognitive load and deliver the right amount of information at the right time, increasing the likelihood of clinician adherence to PGx-based recommendations.

### 3.3. Phenoconversion Calculators and Combined Genotype Prediction Models

A concept that complicates PGx interpretation is “*phenoconversion*,” a phenomenon wherein certain environmental factors (e.g., drug–drug interactions) alter a patient’s effective drug-metabolizing phenotype. For example, if a patient who has CYP2D6 normal metabolizer status and their pain has been well-controlled on tramadol now gets prescribed paroxetine (a strong CYP2D6 inhibitor) for depression. Phenoconversion may occur and tramadol may no longer be an effective analgesic for this patient similar to a patient with CYP2D6 poor metabolizer status. As patients with multiple comorbidities, critical illnesses, or those undergoing surgical procedures may be prescribed multiple new medications, it can become challenging for clinicians to manually monitor for phenoconversion in real-time (or have the required knowledge to do this effectively). A recent systematic review reported that common causes for phenoconversion included concomitant use of CYP450-inhibiting drugs, increasing age, cancer, and inflammation [64]. In addition, use of CYP450 inducers, smoking, alcohol consumption, pregnancy, and vitamin D exposure were suggested as factors that could result in higher metabolizer status [64].

In some HIV-positive patients, CYP2D6 activity approaches that of poor metabolizers, despite having a normal metabolizer genotype [65]. Jones et al. reported that compared with age- and sex-matched healthy volunteers, HIV-infected subjects had 90% lower CYP2D6 activity, 53% lower N-acetyltransferase-2 (NAT2) activity and 18% lower hepatic CYP3A4 activity [65].

Automated calculators that integrate genotype with the enzyme activity based on the genotype (activity score) can better approximate a patient’s phenotypic status. Such tools enhance precision in dose recommendations and reduce under- or over-treatment while minimizing cognitive load for clinicians [66]. They can especially be valuable for patients taking numerous drugs such as elderly populations, chronic pain, complex cardiology or oncology patients.

Given the fact that a single drug may be influenced by several different pharmacogenes, an informatics approach which models influence of various genotypes to provide tailored recommendations, is also beneficial. In one study, a model which considered combined genotype resulted in revised categorical risk level (high versus low) calculated based on single gene effects for 41.5% of the subjects [67].

### 3.4. Increased Genomic Diversity and PGx Integration into the Drug Development Pipeline

Our understanding of variants is largely informed by European populations. 17% of the UK Biobank population have at least one undocumented deleterious variant out of the 14 analyzed PGx genes, most of these being of non-European ancestry [68]. Moreover, 72% of individuals enrolled in clinical trials leading to US Food and Drug Administration (FDA) drug approval were of Caucasian descent in 2019 [1]. Biased and underpowered, these studies fail to estimate or underappreciate the strength of severe PGx interactions involving variants mainly seen in minority populations.

Critically, there is a lack of Asian representation, even though they comprise 60.4% of the global population [69]. As of September 2024, the total composition of individuals in the Genome Wide Association Studies Diversity Monitor had less than 1% representation from all ancestries aside from Asian (3.96%) and European (94.48%) [24].

The FDA approval of drugs has no set minimum standards for diversity in clinical trials, merely the requirement of a diversity management plan [70,71]. Moreover, the FDA fails to adequately define diversity, limiting implementation within trials [70,71]. Given many adverse effects may be explained by PGx, implementing baseline PGx testing across drug trials could be an evidence-based measure to conceptualize diversity. Thereby helping identify any biases in genetic representation within trial cohorts and drawing attention to address any genotype gaps before drugs are introduced to new demographics [72].

Future efforts to engage more diverse groups into trials is vital as emerging technologies seek to accelerate drug development through conducting preliminary testing “in silico”, using available datasets. We note that the NIH All of Us Research Program will be a great step forward for PGx, through collecting data via whole genome sequencing, and microarray data across a diverse >1 million participants with linked EMR information. This data will especially benefit for patients with mixed race ancestry and provide opportunities to identify new pharmacogenes.

### 3.5. Prioritization of High Yield Fields and Groups

Identifying which patient populations benefit the most from panels remains a key part of PGx optimization. Oncology (for cytotoxic and targeted therapy dosing, certain anti-emetics), solid organ transplant (for dosing tacrolimus), cardiovascular medicine and neurology (antiplatelet therapy efficacy and statin myopathy risks), anesthesiology (halogenated volatile anesthetics or succinylcholine and risk of malignant hyperthermia with certain genotypes), and psychiatry and pain medicine (anti-depressants and select opioids) are key targets [73]. Testing for CYP2C19 metabolizer status prior to clopidogrel commencement for acute coronary syndrome patients, is supported by the American Heart Association and demonstrated to significantly decrease the risk of recurrent myocardial infarction [74,75,76]. The perioperative period presents a unique opportunity to utilize PGx as most patients undergo comprehensive medical assessment prior to elective procedures, many new medications are prescribed for the surgical procedure and for management of any new medical complications or conditions that may occur in the perioperative period.

Implementing PGx tailored care could be prioritized in these fields to more clearly demonstrate the benefits of PGx, in currently available guidelines, which can then provide rationale to expand more widely across clinical disciplines. Given the cost of many oncology agents, a case can especially be made for the cost effectiveness of PGx tailored prescribing. In addition, individuals with chronic conditions including rheumatologic diseases (which is especially under-researched) and chronic pain, stand to accrue significant long-term benefits from PGx throughout their life course.

These groups can be further focused to ethnicity specific cohorts, as some ancestries may have greater prevalence of abnormal pharmacogenes. In a South Korean PGx sample, Clopidogrel prescription required prescription changes with 62.4% exhibiting a CYP2C19 phenotype contraindicating usage in both cardiovascular and neurovascular treatment regimens [77].

Enfuvirtide (for AIDS treatment, required dosage adjustments in 34.8% of individuals, and Tacrolimus, (crucial for post-transplant immune rejection prevention), necessitated dosage increases beyond standard doses for 42.4% of participants. For statin therapy, aimed at managing hyperlipidemia, lovastatin and simvastatin were found to be unsuitable for 22.5% of the sample studied [77].

## 4. Conclusions

PGx stands at an inflection point: it has proven its usefulness in multiple high-acuity therapeutic areas, yet it remains underutilized due to challenges in reimbursement, inconsistent guideline application, and the over-reporting of non-actionable data. Moving forward, a concerted effort by researchers, clinicians, and payers is necessary to streamline PGx offerings. This must be met with provider education on how to implement PGx panel testing into consultations and be aware of its limitations. Continued discovery of new pharmacogenes and alleles, in addition to upgrading CPIC B and C guidelines into A ratings (based on evidence from larger association studies), will enhance the value proposition of PGx.

Reports must be concise and include the most clinically relevant information, supported by just-in-time alerts that are integrated with EMR systems. As Medicare and Medicaid lead the way toward broader coverage, commercial payers are likely to follow suit. Ultimately, the future of PGx may have to rely on insurance companies as opposed to physicians being the ones who recommend and take charge in terms of making it available for patients. Health systems instead of individual prescribers may need to drive widespread adoption, due to the multifactorial system changes needed, ranging from pharmacist PGx consulting services to clinician education. The challenges raised by allele gaps, including metabolizer category limitations and assigning responsibility for consequent ADRs from mis-informed prescribing, must be addressed as they risk characterizing PGx as more effort than it is worth.

Future research should explore LLMs as a means to provide real-time decision support that adapts to new evidence, while autotuning phenotype calculations for improved dosing accuracy. By focusing on practical implementation, clinical outcomes, and validated economic impact, PGx can achieve widespread adoption and bring personalized care to patients.

As a clinical community we must promote the benefits of PGx by highlighting cases where PGx tailored prescribing has led to decreased side effects and enhanced trust/patient satisfaction and follow up attendance. The integration of pharmacogenomics (PGx) into routine clinical practice necessitates provider, pharmacist, and clinician investment from across the entire health care value chain.

## Data Availability

No new data were created or analyzed in this study. Data sharing is not applicable to this article.

## Abbreviations

The following abbreviations are used in this manuscript:

PGx	Pharmacogenomics
CPIC	Clinical Pharmacogenetics Implementation Consortium
DWPG	Dutch Pharmacogenetics Working Group
IM	Intermediate Metabolizer
PGx	Pharmacogenomics
PM	Poor Metabolizer
UM	Ultra-Rapid Metabolizer
FDA	U.S. Food and Drug Administration
AMP	Association for Molecular Pathology
EMR	Electronic Medical Record
INR	International Normalized Ratio
LLMs	Large Language Models
CDS	Clinical Decision Support
QALYs	Quality-Adjusted Life Years
CPT	Current Procedural Terminology
NHS	National Health Service
NIH	National Institutes of Health
GWAS	Genome-Wide Association Studies
IM	Intermediate Metabolizer
UM	Ultra-Rapid Metabolizer
